# Proximal femur prosthetic interposition arthroplasty for painful dislocated hips in children with cerebral palsy

**DOI:** 10.1007/s11832-016-0775-z

**Published:** 2016-10-27

**Authors:** Anthony L. Silverio, Shawn V. Nguyen, John A. Schlechter, Samuel R. Rosenfeld

**Affiliations:** 1 Orthopedic Department, Riverside University Health System, 26520 Cactus Avenue, Moreno Valley, CA 92555 USA; 2 Children’s Hospital of Orange County, Orange, CA USA; 3 Adult & Pediatric Orthopedic Specialists, 1310 West Stewart Drive, Suite 508, Orange, CA 92868 USA

**Keywords:** Cerebral palsy, Interpositional arthroplasty, Hip dislocation, Spasticity, Proximal femur arthroplasty

## Abstract

**Purpose:**

Children with cerebral palsy often have musculoskeletal disorders involving the hip. There are several procedures that are commonly used to treat these disorders. Proximal femur prosthetic interposition arthroplasty (PFIA) is an option for non-ambulatory children with cerebral palsy who have a painful, spastic dislocated hip. The purpose of our study was to evaluate the results of PFIA by examining treatment outcomes, complications, and overall effects on the child and their caregiver.

**Methods:**

Charts were reviewed over a 5-year period at our institution. The focus of the data collection was pain, range of motion (ROM), and overall clinical outcome. Clinical outcome was graded as excellent, good, fair, and poor. Length of follow-up, presence of heterotopic ossification, femoral prosthesis migration, and information provided by competed caregiver questionnaires were analyzed.

**Results:**

A total of 16 hips in 12 patients met the inclusion criteria. Average age at time of surgery was 12 years 1.2 months. Average follow-up was 40.4 months. Three hips required revision surgery. Average time before revision surgery was 16 months. Overall outcomes were excellent/good for seven hips and fair/poor for nine. Pain outcomes were excellent/good for nine hips and fair/good for seven. ROM outcomes were excellent/good for nine hips and fair/poor for seven. The majority of caregivers surveyed would recommend this procedure.

**Conclusion:**

Clinical evaluation of the effectiveness of PFIA yielded variable results with this cohort of children with regards to pain and range of motion. Despite these varied results, the majority of caregivers were satisfied with the outcome and would recommend PFIA. PFIA is a salvage option for the painful, spastic dislocated hip, but significant evidence to prove its effectiveness over other salvage procedures is lacking. Based on our results, we conclude that PFIA has the ability to benefit children with cerebral palsy with an acceptable risk profile similar to that reported in recent publications.

Level of evidence IV; retrospective case-series.

## Introduction

Children with cerebral palsy often have musculoskeletal disorders involving the hip [1]. These disorders cover a wide spectrum, ranging from subluxation of the hip at risk to dislocation to dislocation with severe degeneration and pain. Non-ambulatory children with cerebral palsy and increased spasticity tend to develop hip subluxations and subsequent dislocations over time [2]. This leads to progressive erosions and degeneration of the femoral head and/or acetabulum [3, 4]. These children can develop severe pain and often have difficulty with sitting, sleeping, and quality of life [5, 6]. Furthermore, perineal hygiene may be impaired from limitations in hip abduction or pain with motion, thereby increasing the burden of care and attention required from caregivers [6].

Various surgical procedures have been described for children with hip dislocations from a neuromuscular etiology, who may be amenable to a complex reconstructive procedure with potentially high morbidity. Salvage procedures may also be an option for these children and include proximal femur resection arthroplasty (PFRA), re-directional valgus osteotomies with or without femoral head resection, hip arthrodesis, and total hip arthroplasty [7–15]. Alternatively, proximal femur prosthetic interposition arthroplasty (PFIA) is an option for non-ambulatory children with cerebral palsy who have painful, spastic dislocated hips and who are not candidates for reconstruction. This latter group of children is the focus of the investigation reported here [16, 17].

The purpose of our study was to evaluate the results of PFIA by examining treatment outcomes, complications, and overall effects on the child and their caregiver.

## Methods and operative technique

### Methods

**Fig. 1:**
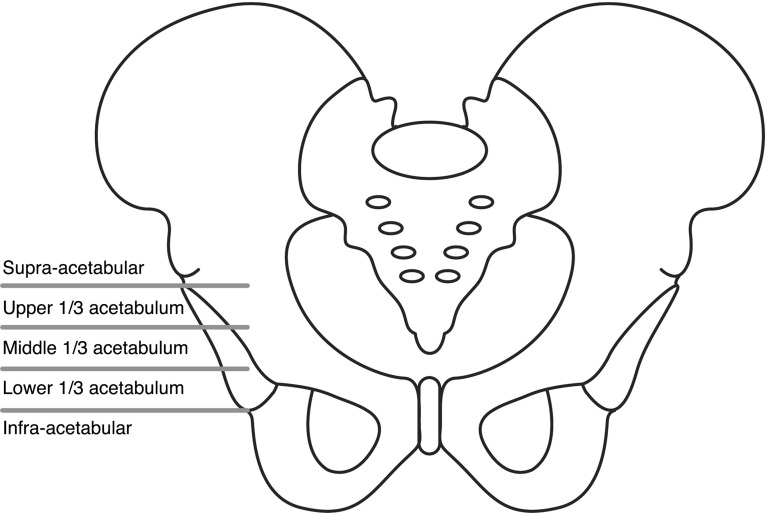
Femoral prosthesis migration relative to the acetabulum which we classified as infra-acetabular, lower 1/3, middle 1/3, upper 1/3 and supra-acetabular on post-operative radiographs

Following Institutional Review Board approval, we conducted a retrospective chart review of children with cerebral palsy who had undergone a PFIA for a spastic, painful dislocated hip from 2007 to 2012. Children were included in this study if they had a diagnosis of cerebral palsy, had undergone a PFIA secondary to a painful, spastic dislocated hip, and had at least 24 months of follow-up, if radiographs were available at the final follow-up, and if a caregiver questionnaire survey had been completed. All children were classified using the Gross Motor Function Classification System [18]. Charts were reviewed for indications of surgery, the need for further surgery, the indications for further surgery, the amount of pain and range of motion (ROM) at final follow-up, the presence and severity of heterotopic ossification (HO) using the Brooker Classification, and the amount of estimated blood loss during the surgery [19]. Prosthesis migration was also measured in two ways: location of prosthesis at time of final follow-up, which was classified as infra-acetabular level [lower one-third, middle one-third, or upper one-third of the acetabulum, and supra-acetabular (Fig. 1)] and average percentage of prosthesis migration at final follow-up. Children were excluded from this study if their follow-up was less than 24 months, or if their clinical outcome could not be graded by the following criteria due to insufficient records.

The outcome of each procedure with respect to the child’s pain level and ROM was recorded using the methods of Wright et al. [17]. These outcomes were noted from each child’s medical record. With respect to pain, in accordance with Wright et al.’s [17] methods, we considered those children without any complaint of pain to have an excellent result; those with pain but without a need for medication, a good result; those with pain which was treated and tolerable after oral analgesic administration, a fair result; those with uncontrollable pain, a poor result [17].

Each child’s ROM was graded using the same values: excellent, good, fair, and poor. Wright et al. [17] defined an excellent result when the child had no restrictions with seating; a good result when there was some loss of motion; a fair result when the child required wheel chair modifications in order to sit; a poor result when wheelchair modifications would not improve the child’s ability to sit [17]. We decided the overall outcome of the procedure as the worse of the two results with respect to pain and ROM.

A questionnaire survey was conducted with the child’s caregiver by means of telephone interviews in order to further assist long-term outcome assessment. These subjective questionnaires were asked no earlier than the most recent follow-up visit. A researcher, who was identified as an independent observer and not part of the surgical team, administered the questionnaire. The child and/or the caregiver were informed that the responses to the questions would be kept confidential from the surgeon and would in no way affect their follow-up schedule. The questions focused on caregivers’ satisfaction with the child’s pain level, sitting tolerance, and perineal care, overall satisfaction, and recommendation of the PFIA to others. Each question was answered on a 0–10 scale in which 0 represented the child’s outcome was much worse since the surgery, 5 represented there was no change since the surgery, and 10 represented there was much improvement since the PFIA.

Statistical analysis was performed using GraphPad PRISM software (GraphPad Software Inc., La Jolla, CA).

#### Operative technique

A lateral approach to the proximal femur was performed in all cases. The average incision length was approximately 14 cm. The deep fascia was opened the length of the incision. A “Y” incision was made over the greater trochanter elevating the gluteus medius, short external rotators and vastus lateralis. The muscles were elevated exposing the hip capsule. These muscles were later repaired in a cerclage fashion over the shoulder prosthesis to limit migration of the implant. The hip capsule was elevated off of the femoral neck and head. After debridement of the joint, a cerclage closure of the hip capsule using a heavy, non-absorbable suture was performed as an interpositional capsular arthroplasty. Next, attention was turned to the proximal femur, where sub-periosteal dissection was carried out to expose at least 2 cm below the lesser trochanter. At this level a transverse cut was made and the proximal femoral segment was liberated and removed. Once this step was completed, the femoral canal was prepared with sequential reaming. An adult shoulder prosthesis (Zimmer, Warsaw, IN) was then chosen. A drill hole was placed in the proximal femur, and a heavy, non-absorbable suture was placed through the drill hole to act as a cerclage suture to aid in securing the prosthesis. The component was then inserted in a press-fit manner and secured. Bone cement was not used. The remaining muscle sleeve was then re-approximated with heavy, non-absorbable sutures, closing the vastus lateralis, gluteus medius and short external rotators over the prosthesis. The post-operative management protocol involved radiographic imaging in the recovery unit, overnight admission, and parenteral antibiotic administration for the first post-operative 24 h. A 12-week course of either oral indomethacin 25 mg three times daily or ibuprofen 10 mg/kg three times daily was administered for the prevention of HO; if the child was dependent on a gastrostomy tube, this medication was administered via gastrostomy tube.

## Results

The initial study cohort comprised 35 hips in 25 children; however, only 16 hips in 12 children met the inclusion criteria of having a PFIA procedure performed and at least 24 months follow-up, radiographs at final follow-up, and a completed caregiver questionnaire. The average follow-up was 40.4 (range 24–60) months. The average age at the time of surgery was 12 years and 1.2 months (range 9–18 years). Nine of the children were male and three were female. All children were classified as level 5 by the Gross Motor Function Classification System. Four children had bilateral affected hips requiring surgery. All children were indicated for surgery due a to spastic, painful hip dislocation affecting hygiene, sitting tolerance, or quality of life. Six of the children had previous hip procedures to treat their symptoms.

Each procedure was performed by one of three pediatric orthopedic surgeons with fellowship training. In general, there were no significant differences in how each surgeon conducted the procedure. Four hips in three children had a posterior spinal fusion performed during the same operation. Average estimated blood loss (EBL) for the PFIA alone was 155 (range 10–300) ml; when the child had concomitant posterior spinal fusion, the average EBL was 2475 (range 700–3200) ml.

Three hips in two children required revision surgery (18.7%). The first child had a bilateral PFIA and required revision secondary to prominent prostheses, and the second child required revision after developing severe pain and osteolysis detected by radiography. The average time period before revision was 16 (range 15–18) months. All revision procedures consisted of complete removal of the prosthesis, and no additional hip procedures were completed at time of removal. Other post-operative complications included: HO (6 patients), periprosthetic fracture (1 patient), pneumonia (1 patient), prolonged intubation (1 patient), and post-operative anemia (3 patients).

Six children had radiographic evidence of HO by the time of final follow-up (50%) with an average Brooker Grading of 1.4. All children who underwent posterior spinal fusion at the same time as the PFIA were given HO prophylaxis as described above and none had HO at final follow-up. Two hips were measured at the infra-acetabular level, two at the lower one-third of the acetabulum, zero at the middle one-third, four at the upper one-third, and eight at the supra-acetabular level. The average percentage of prosthesis migration at final follow-up was 38%, while the average percentage of prosthesis migration in patients requiring revision surgery was 48% (*p* = 0.31).

**Fig. 2:**
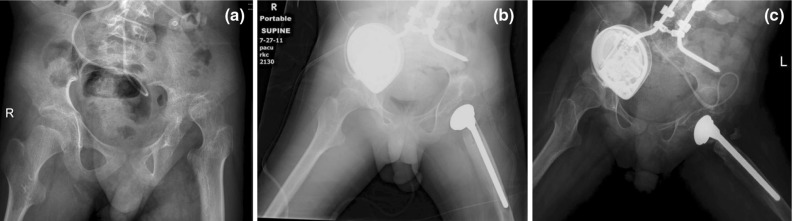
**a** Pre-operative radiograph of a 10-year-old child with cerebral palsy and a left painful, spastic dislocated hip. **b** Immediate post-operative radiograph. **c** Final radiograph at 48 months post-operatively of a child who had an excellent outcome with regards to pain, range of motion (ROM), and overall outcome

**Fig. 3:**
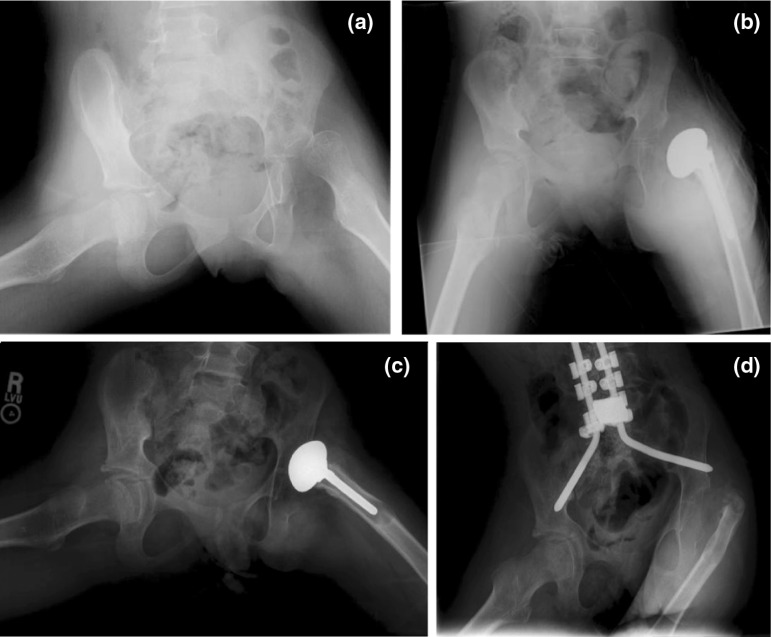
**a** Pre-operative radiograph of a 9-year-old child who required revision surgery 15 months after proximal femur prosthetic interpositional arthroplasty (PFIA) secondary to severe pain and osteolysis. **b** Immediate post-operative radiograph after PFIA. **c** 11-month post-operative radiograph showing osteolysis surrounding the proximal femoral prosthesis. **d** Radiograph showing complete prosthesis removal 15 months after PFIA. Concomitant posterior spinal fusion with instrumentation was completed at time of prosthesis removal

The pain, ROM, and overall outcomes are summarized in Table 1. The overall outcome for the PFIA included 6 excellent (Fig. 2a–c), 1 good, 6 fair, and 3 poor. Of the three poor results, all underwent a revision surgery, which involved removing the prosthesis (Fig. 3a–d). When the results of the procedure were analyzed using ROM as the sole measured outcome, there were six scores of excellent, three good, six fair, and one poor. When pain was the sole outcome measure, there were nine scores of excellent, zero good, four fair, and three poor.

**Table 1:** Outcomes of the proximal femur prosthetic interpositional arthroplasty based on pain, range of motion, and overall outcome

Outcome of PFIA^a^	Pain	Range of motion	Overall outcome^b^
Excellent—Without pain, no restrictions with seating	9	6	6
Good—Pain without need for medication and some loss of motion demonstrated	0	3	1
Fair—Tolerable pain after oral analgesic administration and required wheel chair modifications in order to sit	4	6	6
Poor—Uncontrollable pain and wheelchair modifications would not improve ability to sit	3	1	3

*PFIA* Proximal femur prosthetic interpositional arthroplasty

Values in table are the number of hips in that category

^a^The outcome of each procedure with respect to the child’s pain level and range of motion (ROM) was recorded using the methods of Wright et al. [17]

^b^Decided as the worse of two results with respect to pain and ROM

Table 2 presents the pain, ROM, and overall outcomes for revision surgery. The overall outcome of these cases included two excellent, one good, zero fair, and zero poor. The ROM improvement outcome included two excellent, one good, zero fair, and zero poor. The pain improvement outcome included two excellent, one good, zero fair, and zero poor.

**Table 2:** Outcomes of revision surgery based on pain, range of motion, and overall outcome

Outcome of revision surgery^a^	Pain	Range of motion	Overall outcome^b^
Excellent—Without pain, no restrictions with seating	2	2	2
Good—Pain without need for medication and some loss of motion demonstrated	1	1	1
Fair—Tolerable pain after oral analgesic administration and required wheel chair modifications in order to sit	0	0	0
Poor—Uncontrollable pain and wheelchair modifications would not improve ability to sit	0	0	0

Values in table are the number of hips in that category

^a^The outcome of each procedure with respect to the child’s pain level and ROM was recorded using the methods of Wright et al. [17]

^b^Decided as the worse of two results with respect to pain and ROM

All of the children’s caregivers were available to answer the questionnaire. The summary of the results is listed in Table 3. Using the scale described in the Methods section [range 0 (much worse since the PFIA) to 10 (much improved since PFIA)], the average change in pain was 8.2/10 (range 3–10)m while the average change in sitting tolerance was 6.6/10 (range 1–10). The average change in perineal hygiene was 6/10 (range 5–10). The caretakers’ average satisfaction was 8.6/10 (range 4–10), and their average recommendation was 8.5/10 (range 1–10). Of the two caretakers involved with a child needing revision surgery, one would not recommend this surgery even after revision. All remaining caregivers of children not needing revision surgery would recommend the PFIA.

**Table 3:** Average results from caretakers’ questionnaire surveys

Results of questionnaire survey^a^	Pain	Sitting	Hygiene	Satisfaction	Recommendation
Average score	8.2	6.6	6	8.6	8.5
Range	3–10	1–10	5–10	4–10	1–10

^a^Each question was answered on a 0–10 scale in which 0 represented the child’s outcome was much worse since the surgery, 5 represented there was no change since the surgery, and 10 represented there was much improvement since the PFIA

Statistical analysis was performed to compare those children who only underwent a primary procedure with those who underwent revision according to age (*p* = 0.06), history of previous hip surgery (*p* = 0.40), and amount of prosthesis migration (*p* = 0.31). Each comparison did not yield statistically significant results.

## Discussion

The appropriate treatment of painful, spastic hip dislocations in children with cerebral palsy, who are not candidates for reconstruction, has been an ongoing topic of discussion. Many studies on various methods have been conducted to investigate which treatment provides the most benefit to these children. Such procedures include PFRA, PFIA, subtrochanteric valgus osteotomy (SVO) with or without femoral head resection, hip arthrodesis, and total hip arthroplasty [7–17]. In this context, we have evaluated the effectiveness of PFIA utilizing a shoulder prosthesis. Although based on our clinical assessment the results of the PFIA were variable with regards to pain and ROM, according to our questionnaire results the majority of caregivers were satisfied with the outcome and would recommend PFIA.

Hwang et al. [9] reported on the functional outcomes of salvage procedures for children with cerebral palsy who had chronic dislocations of the hip using validated scoring systems. In their study, the children were divided into three groups: the PFRA group as described by McCarthy et al. [12], the SVO group, and the SVO with resection of the femoral head group [18]. Hwang et al. [9] concluded that these salvage procedures produced similar results; however, they recommended the use of PFRA as the complications are relatively less severe [9]. In comparison to Hwang et al.’s study, we used a shoulder prosthesis as opposed to muscle interposition. Our rationale for use of a shoulder prosthesis is that it prevents bony impingement of the proximal femur on the ilium when proximal migration occurs by acting as an interpositional material.

HO occasionally develops in children with cerebral palsy [20], but the incidence of HO developing in children with cerebral palsy after bony procedures around the hip is still unknown [21]. Over a 5-year span, Tirelli et al. [22] observed HO four times in 39 children who underwent mainly soft tissue releases because of flexion and adduction contractures of the hip, while Krum and Miller [23] reported that the incidence of HO in their cohort was 34% when a child underwent a soft tissue procedure around the hip. Furthermore, these latter authors reported that when a soft tissue procedure around the hip was combined with a posterior spinal fusion the incidence of HO was 40%. In our study, 50% of the children developed HO. Of note, no child who underwent concomitant posterior spinal fusion developed HO.

**Fig. 4:**
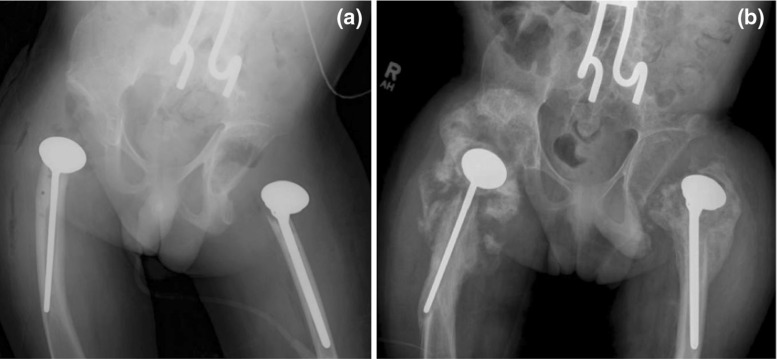
**a** Immediate post-operative radiograph of a child who underwent bilateral PFIA. **b** 25-month post-operative radiograph showing heterotopic ossification of the hips; however, the HO was not clinically significant and the child has yet to require revision surgery

Hwang et al. [9] reported that asymptomatic HO occurred in three patients of their pediatric cohort, but they did not mention whether they employed post-operative HO prophylaxis and, if they did, what the protocol consisted of. Six of the children in our study sustained HO (50%) with an average Brooker grading of 1.4 (Fig. 4a, b); none required revision surgery. In addition, we report our HO prophylaxis protocol. It should be noted that compliance with ibuprofen or indomethacin for 3 months post-operatively was not specifically studied in our cohort.

In another recent article, Patel et al. [14] describe their experience with 20 cerebral palsy patients with painful chronic hip dislocation using a surgical technique that included an augmented interposition myoplasty described by McCarthy et al. [12] and tone management, at a mean follow-up of 54 months. These authors concluded that the myoplasty technique with individualized pain/tone management resulted in good outcomes in this cohort of children. In contrast to our study, Patel et al. [14] did not measure proximal femur migration objectively nor did they perform radiographic examinations routinely at the last follow-up. At the final follow-up we had radiographs for 16 hips, which showed that the most common location of the prosthesis was at the supra-acetabular level (50%) and that the middle one-third of the acetabulum was the least common location (0%). In addition, we calculated prosthesis migration at final follow-up from the initial location of the prosthesis immediately post-operative. An interesting finding was that for those children who required a revision procedure, the average migration was 48% from initial post-operative radiographs, while primary cases only had 38% migration (*p* = 0.31).

Wright et al. [17] compared the clinical outcomes of PFIA with those of PFRA and SVO, two alternative treatments for children with cerebral palsy with a painful dislocated hip, and concluded that the PFIA procedure had the best clinical outcome. However, their study was underpowered and the results are without statistical significance. In order to be able to accurately compare clinical outcomes, we modeled our study design after that of Wright et al. [17]. The notable difference in results between the two studies is that Wright et al. achieved an overall excellent or good outcome in 73% of hips treated with PFIA compared to 44% of hips in our study. Interestingly, in Wright et al.’s study seven of the 11 hips undergoing PFIA required revision surgery compared to three of our 16 hips. We strengthened our study by increasing the sample size, which consisted of 16 PFIA procedures in contrast to 11 in Wright et al.’s cohort. We also evaluated additional post-operative complications that were not mentioned in their study.

Our study is not without its limitations, one of which is its retrospective design. A single researcher performed all data collection in a uniform and detailed manner in order to ensure consistency of assessments. The standardized questionnaire employed during the telephone interviews has not been validated in a separate study. The cases were performed by one of three pediatric orthopedic surgeons with fellowship training and, consequently, the operative technique and post-operative protocols may not be completely standardized. The limited number of children did not allow us to obtain statistical significance in our analysis. Improvements on future related studies could include a multicenter, prospective cohort study design and large numbers of patients in order to conduct valid statistical analysis. More focus could be spent on obtaining assessments from the caregivers since they are more knowledgeable of these children’s everyday health and activity.

Children with cerebral palsy have a multitude of various medical problems. From an orthopedic perspective, one of the most common complaints is a painful, spastic dislocated hip that affects their daily activities and quality of life. Although various treatments are currently used today, a gold standard method has yet to be elucidated. PFIA is a salvage option for the painful, spastic dislocated hip, but significant evidence to prove effectiveness over other salvage procedures is lacking. Based on our results, we conclude that a PFIA may have the ability to benefit children with cerebral palsy with an acceptable risk profile similar to that of recently published studies.
